# *In vitro* characterization of a pAgo nuclease *Ttd*Ago from *Thermococcus thioreducens* and evaluation of its effect *in vivo*

**DOI:** 10.3389/fbioe.2023.1142637

**Published:** 2023-03-02

**Authors:** Ying Tang, Fei Wang, Yi Wang, Yuwei Wang, Yang Liu, Zhizhao Chen, Wenqiang Li, Shihui Yang, Lixin Ma

**Affiliations:** State Key Laboratory of Biocatalysis and Enzyme Engineering, School of Life Sciences, Hubei University, Wuhan, China

**Keywords:** prokaryotic Argonaute, *Thermococcus thioreducens*, DNA-guided DNA endonuclease, genome editing, *Zymomonas mobilis*, cell toxicity

## Abstract

In spite of the development of genome-editing tools using CRISPR–Cas systems, highly efficient and effective genome-editing tools are still needed that use novel programmable nucleases such as Argonaute (Ago) proteins to accelerate the construction of microbial cell factories. In this study, a prokaryotic Ago (pAgo) from a hyperthermophilic archaeon *Thermococcus thioreducens* (*Ttd*Ago) was characterized *in vitro*. Our results showed that *Ttd*Ago has a typical DNA-guided DNA endonuclease activity, and the efficiency and accuracy of cleavage are modulated by temperature, divalent ions, and the phosphorylation and length of gDNAs and their complementarity to the DNA targets. *Ttd*Ago can utilize 5′-phosphorylated (5′-P) or 5′- hydroxylated (5′-OH) DNA guides to cleave single-stranded DNA (ssDNA) at temperatures ranging from 30°C to 95°C in the presence of Mn^2+^ or Mg^2+^ and displayed no obvious preference for the 5′-end-nucleotide of the guide. In addition, single-nucleotide mismatches had little effects on cleavage efficiency, except for mismatches at position 4 or 8 that dramatically reduced target cleavage. Moreover, *Ttd*Ago performed programmable cleavage of double-stranded DNA at 75°C. We further introduced *Ttd*Ago into an industrial ethanologenic bacterium *Zymomonas mobilis* to evaluate its effect *in vivo*. Our preliminary results indicated that *Ttd*Ago showed cell toxicity toward *Z. mobilis*, resulting in a reduced growth rate and final biomass. In conclusion, we characterized *Ttd*Ago *in vitro* and investigated its effect on *Z. mobilis* in this study, which lays a foundation to develop Ago-based genome-editing tools for recalcitrant industrial microorganisms in the future.

## Introduction

To meet the immediate challenges of the energy crisis and global climate change, it is crucial to accelerate the development of microbial cell factories for carbon-neutral biofuel and biochemical production, which calls for effective and efficient genome-editing tools for recalcitrant industrial microorganisms. Although CRISPR–Cas tools including the endogenous Type I-F CRISPR–Cas system and exogenous CRISPR–Cas12a system have been established for industrial microorganisms such as *Zymomonas mobilis* and applied to genome editing processes, such as gene deletion and replacement, *in situ* modifications, and simultaneous multiple gene editing (Cao et al., 2017; Shen et al., 2019; Zheng et al., 2019; Banta et al., 2020; Wang X. et al., 2021; Sui et al., 2021), highly efficient and effective genome-editing tools are still needed to accelerate the construction of microbial cell factories using recalcitrant non-model microorganisms such as *Z. mobilis*.

The conserved programmable Argonaute (Ago) nuclease proteins exist widely in eukaryotes and prokaryotes (Swarts et al., 2014b) and have attracted extensive attention for their development as genome-editing tools. Most Agos from prokaryotes (pAgos) are derived from thermophilic organisms, which utilize DNA guides to recognize and cleave complementary DNA or RNA targets; examples include *Aa*Ago, *Tt*Ago, *Pf*Ago, *Mp*Ago, *Mj*Ago, *Fp*Ago, and *Ttr*Ago (Swarts et al., 2014a; Swarts et al., 2015; Willkomm et al., 2017; Zander et al., 2017; Guo et al., 2021). In addition, a few pAgos from mesophilic or psychrotolerant microorganisms have also been characterized such as *Km*Ago, *Cb*Ago, *Lr*Ago, and *Mbp*Ago (Gibson et al., 2009; Enghiad & Zhao, 2017; Hegge et al., 2019; Kuzmenko et al., 2019; Liu Y. et al., 2021; Li W. et al., 2022). Compared with Cas nucleases, Ago proteins do not need additional sequences such as PAMs for target cleavage. Due to the programmability and precise recognition capacity, some pAgos have been applied for the cleavage and detection of nucleic acids (Wang et al., 2009; Enghiad & Zhao, 2017; He et al., 2019; Liu Q. et al., 2021; Wang L. et al., 2021; He et al., 2021; Li W. et al., 2022; Zeng et al., 2022). However, to the best of our knowledge, there is no report yet to apply it in microorganisms for developing Ago-based genome-editing tools.

*Z. mobilis* is generally regarded as safe (GRAS) ethanologenic bacterium with desirable industrial characteristics such as a broad range of pH (pH 3.5–7.5) and temperature (24–40°C) as well as high sugar uptake and high ethanol tolerance and productivity (Rogers et al., 2007; Yang et al., 2016a; Wang et al., 2018; Li et al., 2021), which has been developing as a chassis for the production of carbon-neutral lignocellulosic biofuels and biochemicals including 2,3-butanediol, isobutanol, lactate, and PHB (Yang et al., 2016b; Liu et al., 2020; Qiu et al., 2020; Li Y. et al., 2022).

In this study, a pAgo from the hyperthermophilic archaeon *Thermococcus thioreducens* (*Ttd*Ago) was characterized *in vitro*, and the impact of introduction of pAgo into *Z. mobilis* was investigated for the first time*.*

## Materials and methods

### Strains, media, and growth conditions

In this study, *Z. mobilis* ZM4 (ATCC 31821) was used as the parental strain and cultured at 30°C with shaking at 100 rpm in RM medium (50 g/L glucose, 10 g/L yeast extract, 2 g/L KH_2_PO_4_, and 1.5% agar for solid). *E*. *coli* DH5α was used for plasmid construction, and all *E. coli* strains were cultured in Luria–Bertani (LB) medium (10 g/L tryptone, 5 g/L yeast extract, 10 g/L NaCl, and 1.5% agar for solid) at 37°C, 250 rpm. When required, 100 or 300 μg/mL kanamycin was added to *E*. *coli* or *Z. mobilis*, respectively.

### Multiple sequence alignment and phylogenetic tree analysis

The nucleotide sequence of the *TtdAgo* gene (WP_055429304.1; *Thermococcus thioreducens*) was retrieved from the NCBI database. Online software Clustal Omega (https://www.ebi.ac.uk/Tools/msa/clustalo/) was used to compare some of the currently studied Ago proteins for multiple sequence comparisons. To further analyze the affinities of TtdAgo with other studied Ago proteins, phylogenetic tree analysis was performed by using the maximum likelihood method using MEGA X software based on the results of multiple sequence alignment.

### Protein expression and purification

The nucleotide sequences of the *Ttd*Ago and *Ttd*Ago_DM genes (D538A and D608A) were codon-optimized for expression in *E. coli*. The *Ttd*Ago and *Ttd*Ago_DM genes were synthesized (GeneCreate Biotechnology, Wuhan, China) and cloned into a pET23a expression vector in frame with the C-terminal ×6 His tag. The *Ttd*Ago and *Ttd*Ago_DM proteins were expressed in *E. coli* Rosetta (DE3) (Novagen, Darmstadt, Germany). Cultures were grown at 37°C in LB medium containing 50 μg/mL ampicillin induced by adding isopropyl-β-D-1-thiogalactopyranoside (IPTG) to a final concentration of 0.5 mM until OD_600_ reached 0.8. During the expression, cells were incubated at 18°C for 20 h with continuous shaking. The cells were collected by centrifugation and stored at −80°C for further protein purification.

The cell pellets were resuspended in Buffer A (20 mM Tris-HCl pH 7.5, 500 mM NaCl, and 10 mM imidazole) supplemented with an EDTA-free protease inhibitor cocktail tablet (Roche, Shanghai, China) and disrupted by sonication (Scientz-IID: 350 W, 2 s on/4 s off for 30 min). The lysates were clarified by centrifugation at 21,000 *g* for 20 min, and the supernatant was loaded onto Ni-NTA agarose resin at 4°C for 1–2 h with rotation and subsequently extensively washed with Buffer A containing 50 mM imidazole. The bound protein was eluted with Buffer A containing 300 mM imidazole. The eluted protein was concentrated against Buffer B (20 mM HEPES pH 7.5, 500 mM NaCl, and 1 mM dithiothreitol (DTT)) by ultrafiltration using an Amicon 50K filter unit (Millipore, United States). Next, the protein was diluted in 20 mM HEPES pH 7.5 to lower the final salt concentration to 125 mM NaCl. The diluted protein was applied to a heparin column (GE Healthcare, Boston, United States of America) and equilibrated with Buffer C (20 mM HEPES pH 7.5, 125 mM NaCl, and 1 mM DTT), then washed with at least 10 column volumes of the same buffer and eluted with a linear NaCl gradient (0.125–1 M). Fractions containing *Ttd*Ago were concentrated by ultrafiltration using an Amicon 50K filter unit and purified on a Superdex 200 16/600 column (GE Healthcare, Boston, United States). The protein was eluted with Buffer B (20 mM HEPES pH 7.5, 500 mM NaCl, and 1 mM DTT). Purified *Ttd*Ago was concentrated using an Amicon 50K filter unit and diluted in Buffer B to a final concentration of 8 μM, aliquoted, and flash-frozen in liquid nitrogen. The purified protein was stored at −80°C.

### Single-stranded nucleic acid cleavage assays

Cleavage assays were performed using synthetic guides and targets. Most reactions were performed with pAgo, guide, and target at the molar ratio of 4:4:1. A measure of 800 nM *Ttd*Ago was mixed with 400 nM gDNA or gRNA and incubated for 10 min at 37°C using a PCR thermocycler (T100, Bio-Rad, CA, United States) for guide loading in buffer RB (10 mM HEPES pH 7.5, 100 mM NaCl, and 5% glycerol) with 5 mM MnCl_2_. Then added 200 nM of nucleic acid target. The reactions were performed in PCR tubes at 75°C for 20 min and stopped after indicated time intervals by mixing the samples with the ×2 RNA loading dye (95% formamide, 18 mM EDTA, 0.025% SDS, and 0.025% bromophenol blue) and heating it for 5 min at 95°C. The cleavage products were resolved by 20% denaturing PAGE, stained with SYBR Gold (Invitrogen, CA, United States), visualized using Gel DocTM XR+ (Bio-Rad, CA, United States), and analyzed using ImageJ software.

### Double-stranded DNA cleavage activity assay

In two half-reactions, 800 nM *Ttd*Ago was preloaded with either 1,000 nM forward or reverse DNA guide in a reaction buffer containing 5 mM HEPES-NaOH pH 7.5, 100 mM or 250 mM NaCl, 5 mM or 10 mM MgCl_2_, and 2.5% glycerol. The half-reactions were incubated for 30 min at 37°C. Next, both half-reactions were mixed, and 200 ng target plasmid was added. Then, the mixture was incubated for 20 min at 75 °C. A ×6 DNA loading dye (NEB, MA, United States) was added to the plasmid sample prior to resolving it on a 1% agarose gel stained with ethidium bromide.

### Genetic manipulation and recombinant strain construction

The shuttle plasmid pTZ22b was used for *Ttd*Ago expression in *Z. mobilis* ZM4. For the plasmid construction, primers synthesized by Tsingke (Beijing, China) were used for the polymerase chain reaction (PCR) using DNA polymerase (Takara, Japan) to obtain DNA fragments. All plasmids were assembled by the Gibson Assembly method (Gibson et al., 2009). After purification, gene and vector fragments were ligated through the T5 exonuclease (NEB, WA, United States)-dependent DNA assembly method as described previously (Tang et al., 2022). After transformation in *E. coli*, the correct colonies were selected by colony PCR and confirmed by Sanger sequencing (Tsingke Biotechnology, Beijing, China).

### Electroporation transformation and recombinant strain selection

The recombinant plasmid was transformed into *Z. mobilis* competent cells *via* electroporation (0.1-cm electrode gap, 1600 V, 200 Ω, and 25 μF) using a Gene Pulser® (Bio-Rad, CA, United States of America). The correct colonies were selected by colony PCR. Recombinant cells were placed on RM agar plates with 300 μg/mL kanamycin supplementation, and then the plates were stored at −80°C.

### Cell growth analysis

To prepare the seed culture, *Z. mobilis* strains from frozen glycerol stocks were revived in 50-mL flasks containing 40 mL RM medium. After culturing overnight without shaking to the mid-exponential phase, the seed culture was harvested and inoculated in 50-mL shake flasks containing 40 mL RM medium with an initial OD_600_ nm value of 0.1. Cell growth in terms of the absorbance value at 600 nm (OD_600_) was measured using a spectrophotometer (UV-1800, AOE, China) at different time points.

## Results and discussion

### *Ttd*Ago prefers to cleave DNA rather than RNA with gDNAs at high temperatures

**FIGURE 1:**
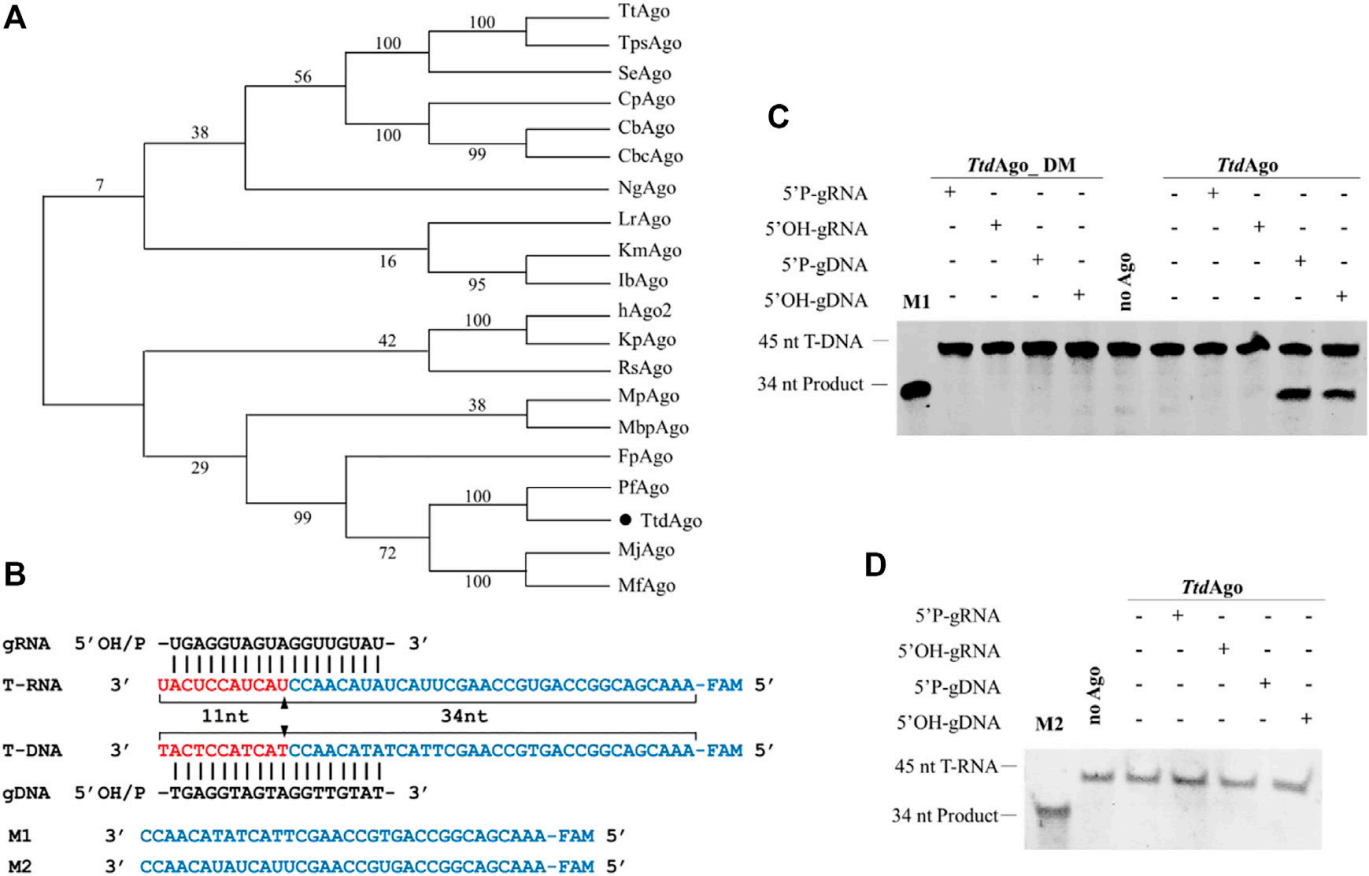
Phylogenetic tree analysis and single-stranded nucleic acid cleavage assays of *Ttd*Ago. **(A)** Maximum likelihood phylogenetic tree of characterized Ago proteins. The numbers at the nodes indicate the bootstrap values for maximum likelihood analysis of 1,000 resampled data sets. **(B)** Sequences of the synthetic let7 miRNA-based guide and target sequences (T-DNA and T-RNA) that were used for the *in vitro* cleavage assays. The black triangles indicate the cleavage site. **(C)** DNA cleavage activity assay of *Ttd*Ago. **(D)** RNA cleavage activity assay of *Ttd*Ago. Positions of the cleavage products are indicated on the left of the gels. *Ttd*Ago, guide, and target were mixed at a molar ratio of 4:4:1 (800 nM *Ttd*Ago preloaded with 800 nM guide, plus 200 nM target) and incubated for 30 min at 75°C. Catalytic dead mutant *Ttd*Ago DM (DM) was used as the control. Lanes M1 and M2 contain chemically synthesized 34-nt DNA and RNA corresponding to the cleavage products of T-DNA and T-RNA, respectively. All reactions were carried out in the reaction buffer containing 5 mM Mn^2+^ ions.

The multiple sequence alignment of *Ttd*Ago with other Agos (Supplementary Figure S1A) suggests that although TtdAgo is phylogenetically closely related to the *Pf*Ago from *Pyrococcus furiosus* (Figure 1A) and contains the canonical catalytic tetrad residues (D538, E576, D608, and H725) in the PIWI domain (Supplementary Figure S1A) that is essential for the nuclease activity (Hegge et al., 2019), *Ttd*Ago (WP_055429304.1) is distantly related to most other characterized eAgos and pAgos with a sequence identity < 20% (Figure 1A; Supplementary Figure S1A). The *Ttd*Ago gene was codon-optimized for expression in *E. coli*, and the catalytically inactive variant of *Ttd*Ago (*Ttd*Ago_DM) was obtained with substitutions of two out of four catalytic tetrad residues (D538A/D608A, Supplementary Figure S1A).

To investigate its biochemical properties and *in vivo* function, *Ttd*Ago and *Ttd*Ago_DM were expressed successfully in *E. coli* using a T7-based pET expression system. The protein was purified using Ni-NTA affinity, heparin column affinity, and size-exclusion chromatography (see Supplementary Figure S1B and Materials and methods for details). We first studied the nucleic acid specificity of *Ttd*Ago with *in vitro* cleavage assay using synthetic oligonucleotides. *Ttd*Ago was preloaded with 18-nt DNA or RNA guides containing a 5′-phosphate (5′-P) or 5′-hydroxyl (5′-OH) group at 37°C for 10 min followed by the addition of complementary 45-nt long ssDNA or RNA targets (Figure 1B). After incubation for 30 min at 75°C in reaction buffer containing 5 mM Mn^2+^, the cleavage products were resolved on 20% denaturing gel and visualized by SYBR Gold staining.

As most pAgos studied strongly prefer to cleave DNA targets (Nakanishi et al., 2012; Lisitskaya et al., 2018; Jolly et al., 2020), *Ttd*Ago can use both 5′-P-gDNA and 5′-OH-gDNA to cleave DNA targets, resulting in the appearance of the 34-nt-long 5′-fragment of the DNA target, and no RNA target cleavage was observed (Figures 1C, D). However, for the guide RNAs, no *Ttd*Ago-mediated cleavage was observed for either DNA or RNA targets, and no cleavage products were observed in the absence of *Ttd*Ago protein or guides (Figures 1C, D). *Ttd*Ago cleavage required the intact catalytic tetrad in the PIWI domain, and point mutations in the tetrad eliminated the activity of *Ttd*Ago (Figures 1C, D).

In summary, similar to previously characterized pAgos from thermophilic prokaryotes such as *Tt*Ago, *Pf*Ago, *Mj*Ago, and *Fp*Ago (Swarts et al., 2014a; Swarts et al., 2015; Willkomm et al., 2017; Zander et al., 2017; Guo et al., 2021), *Ttd*Ago prefers to use DNA guides to cleave DNA targets. This result is similar to a simultaneous study of *Ttr*Ago (*Ttd*Ago) reported earlier by Fang et al. (2022). However, in contrast to most eAgos and pAgos including hAgo2, *Ib*Ago, *Km*Ago, and *Mbp*Ago (Rivas et al., 2005; Liu Y. et al., 2021; Kropocheva et al., 2021; Li W. et al., 2022), whose target cleavage resulted in a shift of the cleavage site mediated by 5′-OH guides compared to that using 5′-P-gDNA as shown by Fang et al. (2022), we did not observe the cleavage position shift for *Ttd*Ago with 5′-P-gDNA compared to that with 5′-OH-gDNA. This result may be due to the different targets and reaction conditions such as the concentration of divalent ion and the temperature both being different. In addition, although Ago proteins do not need the PAM (protospacer adjacent motif) sequence, there were still differences in the efficiency between different targets (Hunt et al., 2021).

### Temperatures and divalent cations affect the cleavage activity of *Ttd*Ago

**FIGURE 2:**
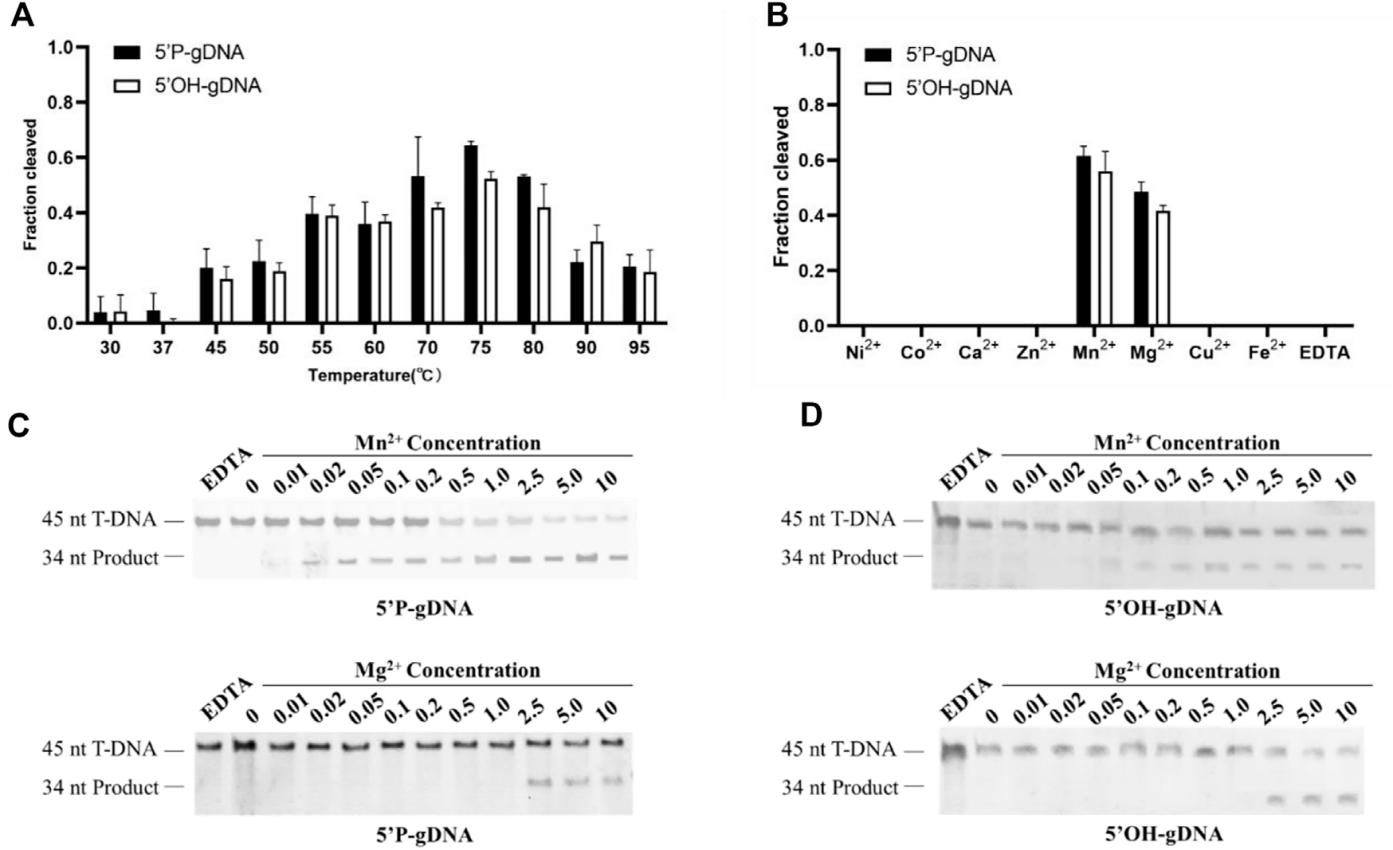
Effects of temperatures and divalent cations on TtdAgo activity. **(A)** Temperature dependence of DNA cleavage by *Ttd*Ago using 5′-P-gDNA and 5′-OH-gDNA. The assay in **(A)** was performed in the reaction buffer containing 5 mM Mn^2+^ ions at indicated temperatures. **(B)** Effects of different cations on DNA cleavage activity mediated by 5′-P-gDNA and 5′-OH-gDNA. The concentration of divalent cations is 5 mM. **(C)** Effects of Mn^2+^ concentration and Mg^2+^ concentration on DNA cleavage activity mediated by 5′-P-gDNA **(D)** Effects of Mn^2+^ and Mg^2+^ concentrations on DNA cleavage activity mediated by 5′-OH-gDNA. The assay in **(B, C, D)** was performed at 75°C for 30 min under different divalent metal ions. All reactions were carried out with *Ttd*Ago, guide, and target at the molar ratio of 4:4:1 (800 nM *Ttd*Ago preloaded with 800 nM guide, plus 200 nM target) with 18-nt guides.

To further determine the prerequisites for *Ttd*Ago-mediated target cleavage, the influence of temperatures and divalent cations on cleavage activity was tested. To explore the active temperature range of *Ttd*Ago, we tested the effect of temperature on DNA cleavage activity mediated by 5′-P-gDNA or 5′-OH-gDNA at temperatures ranging from 30°C to 95°C. The results of the temperature-dependent DNA cleavage activity revealed that *Ttd*Ago bound to 5′-P-gDNA or 5′-OH-gDNA showed activity in the range of 55–95°C, and the best temperature was 75°C (Figure 2A; Supplementary Figure S2A). In summary, the activity of *Ttd*Ago mediated by 5′-P-gDNA or 5′-OH-gDNA exhibited no significant difference between 30°C and 37°C, for example, for 5′-P-gDNA, the cleavage percentage is 0.037 and 0.042, respectively. However, enhanced cleavage activity was displayed from 45°C to 75°C, which then decreased at higher temperatures above 75°C (Figure 2A; Supplementary Figure S2A).

Previous studies also demonstrated that divalent metal ions are crucial for Ago protein activities (Song et al., 2004). Thus, we tested the cleavage activity between 5′-P-DNA and 5′-OH-DNA guides with the DNA target under different divalent metal ions. In the presence of different divalent metal ions (Mg^2+^, Ca^2+^, Mn^2+^, Fe^2+^, Co^2+^, Ni^2+^, Cu^2+^, or Zn^2+^), *Ttd*Ago was active only when Mg^2+^ or Mn^2+^ were used as cations with Mn^2+^ giving a higher activity than Mg^2+^ (Figure 2B; Supplementary Figure S2B). Titration of Mn^2+^ ions showed that *Ttd*Ago was active in the concentration range of 0.02–10 mM and had an increased cleavage activity at Mn^2+^ concentrations ≥ 1.0 mM (Figure 2C). The cleavage efficiency when using 5′-P-gDNA was higher than that when using 5′-OH-gDNA when Mn^2+^ was used, while *Ttd*Ago was active at Mg^2+^ concentrations ≥ 2.5 mM, and the activity exhibited no significant difference between 2.5 and 10 mM no matter whether the guide is 5′-P-gDNA or 5′-OH-gDNA (Figure 2D). Thus, *Ttd*Ago-mediated cleavage was more efficient in the presence of Mn^2+^.

Our study thus demonstrated that *Ttd*Ago cleaved DNA at temperatures ranging from 30°C to 95 °C and had good DNA cleavage activity at 70–80°C, which is consistent with the previous study (Fang et al., 2022). Furthermore, Fang et al. (2022) also found that *Ttd*Ago was active at Mn^2+^ concentrations ≥ 0.1 mM, which is consistent with our results. However, we did not observe effective cutting under Co^2+^, which may be due to the 5 mM Co^2+^ used in this study being much higher than 0.5 mM Co^2+^ used in the previous study. The presence of excess Co^2+^ is likely to inhibit the activity of *Ttd*Ago. In contrast, Fang et al. (2022) did not observe the cleavage product under the presence of Mg^2+^, which may be due to the 0.5 mM Mg^2+^ they used being lower than that required for *Ttd*Ago to be actively functional as we demonstrated that *Ttd*Ago functioned at Mg^2+^ concentrations ≥ 2.5 mM (Figure 2D). These studies thus demonstrated that it is crucial to obtain accurate concentrations of divalent cations for the functionality of pAgos.

### Effects of guide length, concentration, and presence of 5′-P on *Ttd*Ago activity

**FIGURE 3:**
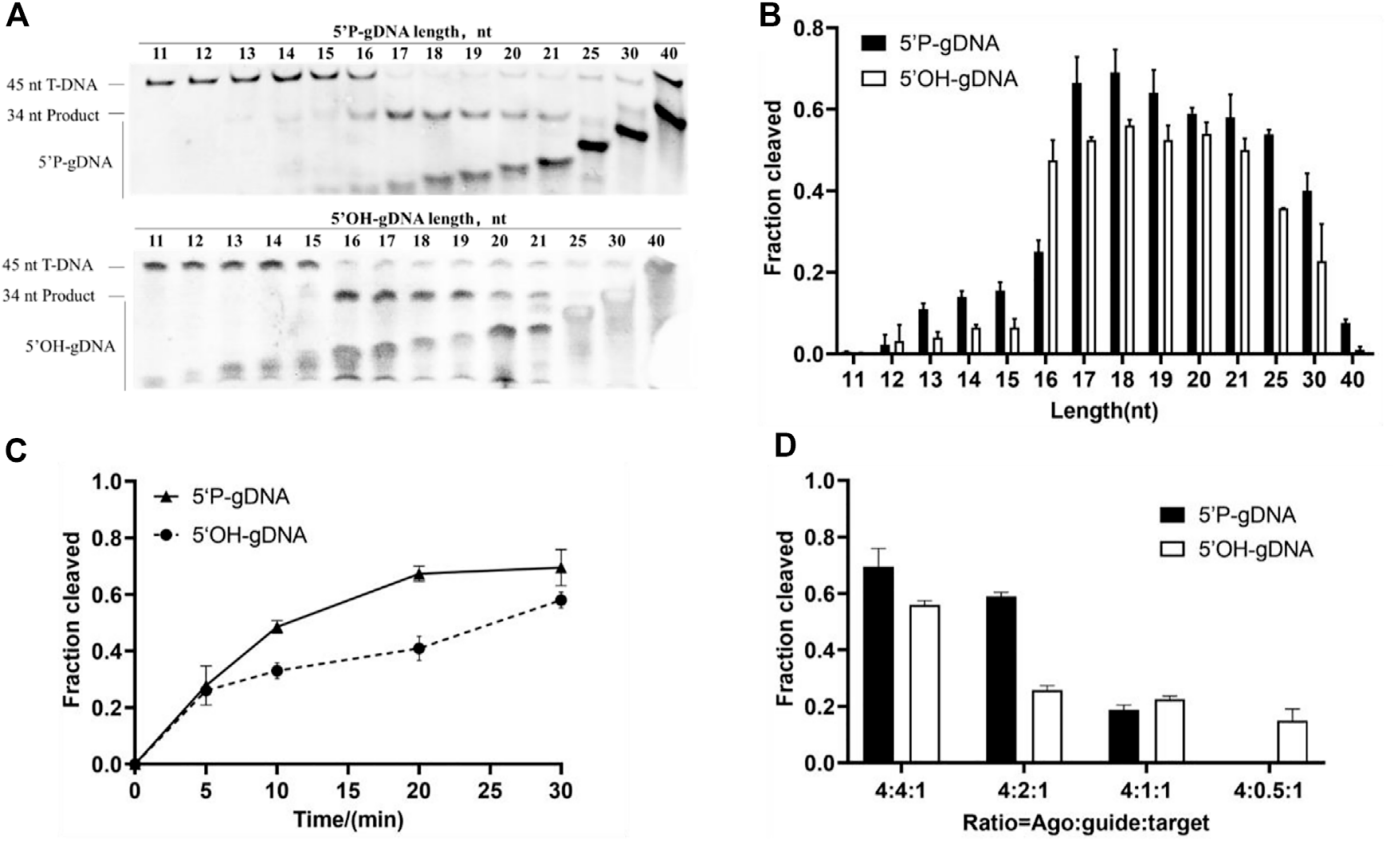
Effects of guide length and concentration on TtdAgo activity. **(A)** Effects of guide length on *Ttd*Ago activity mediated by 5′-P-gDNA and 5′-OH-gDNA. **(B)** Quantification of cleavage efficiencies for **(A)**. The fraction of the cleaved target for each guide length is shown. **(C)** Cleavage kinetics of ssDNA using 5′-P/5′-OH DNA-guided *Ttd*Ago. **(D)** Effects of guide concentration on *Ttd*Ago activity mediated by 5′-P-gDNA and 5′-OH-gDNA. The assay in **(A, B)** was performed using 5′-P-gDNA and 5′-OH-gDNA of different lengths for 30 min. The assay in **(C)** was performed for indicated times (5, 10, 20, or 30 min) with 18-nt guides. The assay in **(A, B, C)** was performed with *Ttd*Ago, guide, and target at the molar ratio of 4:4:1 (800 nM *Ttd*Ago preloaded with 800 nM guide, plus 200 nM target). The assay in **(D)** was performed under different concentrations of guides for 30 min with 18-nt guides. All reactions were carried out in buffer containing 5 mM Mn^2+^ at 75 °C.

Previous studies indicated that the guide length may affect cleavage efficiency (Kropocheva et al., 2021). Thus, we investigated the cleavage activity of *Ttd*Ago using 5′-P-gDNA and 5′-OH-gDNA of different lengths ranging from 11 to 40 nt at 75°C for 30 min with 5 mM Mn^2+^. The cleavage products were observed using 14–40-nt 5′-P-gDNA, and 17–19-nt 5′-P-gDNA was optimal (Figures 3A, B). In the case of 5′-OH-gDNA, *Ttd*Ago was most active using 16–18-nt gDNA with a lower efficiency observed for longer or shorter gDNA (Figures 3A, B). Although we did not observe the cleavage position shift as seen in other Ago proteins reported previously such as *Km*Ago and *Mbp*Ago (Liu Y. et al., 2021; Li W. et al., 2022), the cleavage occurred only between the 10th and 11th guide positions for *Ttd*Ago. In conclusion, according to the relative highest cleavage efficiency of *Ttd*Ago, the most appropriate guide length for both 5′-P-gDNA and 5′-OH-gDNA is 18 nt (Figure 3B). Thus, most experiments were performed using guides with a length of 18 nt subsequently.

Previous studies also suggested that the presence or absence of 5′-P may affect cleavage efficiency (Cao et al., 2019; Kuzmenko et al., 2019). To investigate the catalytic properties of *Ttd*Ago mediated by 5′-P-gDNA and 5′-OH-gDNA, we performed a cleavage kinetics assay at 75°C with 5 mM Mn^2+^. The reaction process of DNA cleavage guided with 5′-P-gDNA was obviously faster than that with 5′-OH-gDNA (Figure 3C). At the beginning of the cleavage reaction, there was no significant difference between these two reactions, but the cleavage percentage mediated by 5′-P-gDNA was higher than that mediated by 5′-OH-gDNA with the extension of the reaction time. For example, when the reaction time was extended to 10 min or 15 min, the cleavage percentages were 0.48 or 0.67 when mediated by 5′-P-gDNA and 0.33 or 0.40 when mediated by 5′-OH-gDNA, respectively. Therefore, *Ttd*Ago prefers to use 5′-P-gDNA as a guide strand.

To evaluate the influence of guide concentration on cleavage activity, the DNA cleavage was monitored under different ratios of *Ttd*Ago, guide, and target using 5′-P-gDNA or 5′-OH-gDNA under the condition of 5 mM Mn^2+^ at 75°C for 30 min. In this experiment, *Ttd*Ago cleavage efficiency was decreased correspondingly when DNA guide supplementation was gradually decreased (Figure 3D and Supplementary Figure S3A). Therefore, most experiments were displayed with *Ttd*Ago, guide, and target at the molar ratio of 4:4:1 (800 nM *Ttd*Ago preloaded with 800 nM guide plus 200 nM target).

Similar to the majority of pAgos, *Ttd*Ago utilizes 5′-phosphorylated DNA guides preferentially. In addition, thermophilic pAgos characterized previously indicate that complementary base pairing of approximately 15 nt between the guide and target is required to form a stable double helix structure at high temperatures. For *Ttd*Ago, a minimum of 16 nt gDNA is required. Furthermore, *Ttd*Ago is most active with a length of 17–19 nt for 5′-P-gDNA and 16–19 nt for 5′-OH-gDNA, with a lower efficiency observed with longer or shorter guides, which is consistent with the previous study (Fang et al., 2022).

### Effects of 5’-end-nucleotide of the guide and guide–target mismatches on *Ttd*Ago activity

**FIGURE 4:**
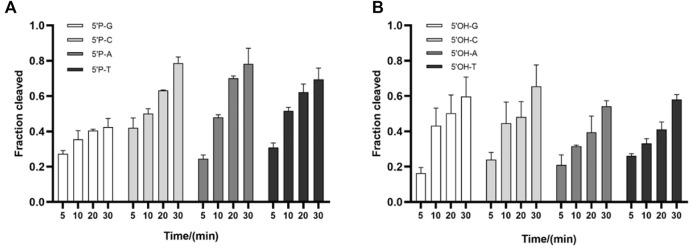
Effects of the 5′-end-nucleotide of the guide and guide–target mismatches on target cleavage. **(A)** Preferences for the 5′-end-nucleotide of the 5′-P-gDNA on DNA cleavage activity. **(B)** Preferences for the 5′-end-nucleotide of the 5′-OH-gDNA on DNA cleavage activity. All experiments were performed with *Ttd*Ago, guide, and target at the molar ratio of 4:4:1 (800 nM *Ttd*Ago preloaded with 800 nM guide, plus 200 nM target) in a reaction buffer containing 5 mM Mn^2+^ at 75°C.

The 5′-end-nucleotide of the guide strand is bound to the MID pocket of Ago proteins, which has certain preference for it (Nakanishi et al., 2012). To determine whether *Ttd*Ago has a bias for the first nucleotide of the guide, cleavage assays were performed using four variants of DNA guides with different 5′-end-nucleotides but otherwise identical sequences. Slightly reduced cleavage rates were observed when *Ttd*Ago loaded with 5′-P-gDNA containing a 5′-G and *Ttd*Ago loaded with 5′-P-gDNA containing 5′-C, 5′-A, or 5′-T cleaved the target comparably (Figure 4A). However, when *Ttd*Ago was loaded with 5′-OH-gDNA with different 5′-end-nucleotides, there were no obvious changes observed for the cleavage efficiency (Figure 4B). In conclusion, in contrast to some thermophilic Agos which have a bias for the first nucleotide of the guides such as *Tps*Ago and *Fp*Ago (Guo et al., 2021; Sun et al., 2022), *Ttd*Ago has no obvious preference for the 5′-end-nucleotide of the DNA guides (Figures 4A, B; Supplementary Figure S3B).

**FIGURE 5:**
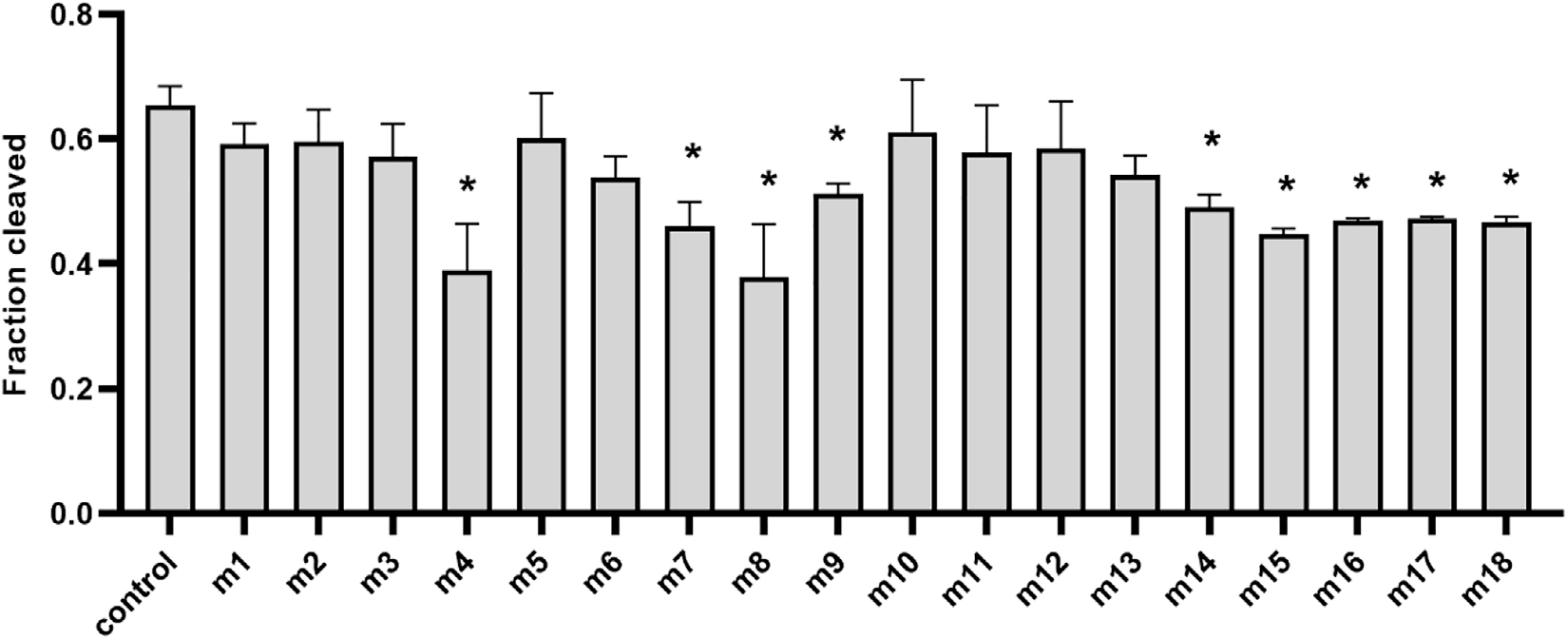
Effects of guide–target mismatches on target cleavage. Data are given as the mean ± SD from three independent measurements. * represents a significant difference (0.01< *p*-value <0.05), and ** represents *p*-value <0.01. The control reaction contained guide without mismatches. The reaction was performed with 18-nt 5′-P-gDNA at 75°C for 30 min, and all experiments were performed with *Ttd*Ago, guide, and target at the molar ratio of 4:4:1 (800 nM *Ttd*Ago preloaded with 800 nM guide, plus 200 nM target) in the reaction buffer containing 5 mM Mn^2+^. Statistical analysis was performed using Student’s t-test.

Previous studies also showed that mismatches between the guide and target may have large interference on the cleavage efficiency and precision of Ago proteins (Dayeh et al., 2018; Liu Y. et al., 2021; Li W. et al., 2022), and even a single mismatch in the seed region (guide positions g2–g8) of the guide can greatly reduce target recognition and cleavage (Miyoshi et al., 2016; Liu et al., 2018). To explore the mismatch tolerance of *Ttd*Ago, we designed a set of DNA guides containing a single-nucleotide or dinucleotide mismatches at certain positions (Supplementary Table S1) to investigate the DNA cleavage activity with *Ttd*Ago (Figure 5, Supplementary Figure S4). When a single-nucleotide mismatch was introduced, such as at positions 4, 7–9, or 14–18, mismatches at most positions affected the cleavage efficiency but did not lead to a dramatic decrease in cleavage efficiency, except for mismatches at positions 4 and 8 with cleavage efficiency reduced by 50%. In summary, *Ttd*Ago has a high tolerance for mismatches between the guide and target strands.

It has been demonstrated that thermophilic Agos such as *Tps*Ago and *Fp*Ago had biases for the first nucleotide of the guides (Cao et al., 2019; Guo et al., 2021). However, *Ttd*Ago has no obvious preference for the 5′-end-nucleotide of a guide, which is similar to other pAgos such as *Km*Ago and *Mbp*Ago (Liu Y. et al., 2021; Li W. et al., 2022). Previous studies revealed the significance of complementarity between the guide and target for Ago cleavage. *Ttd*Ago has a high tolerance for mismatches between the guide and target strands. Therefore, *Ttd*Ago can potentially be used to clear DNA virus because it is difficult for the virus to escape by mutating single bases.

### *Ttd*Ago generates double-stranded DNA breaks in double-stranded DNA

**FIGURE 6:**
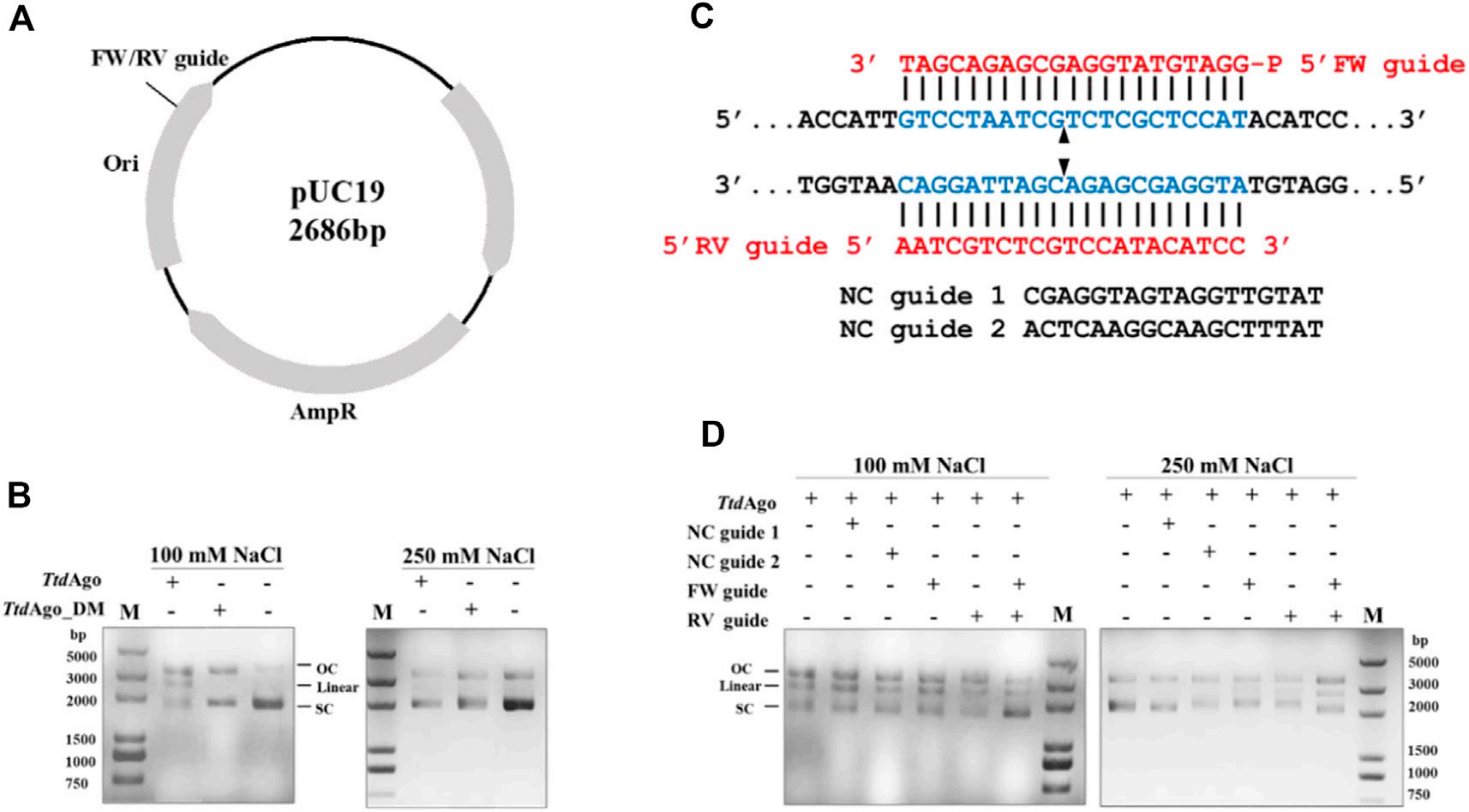
Double-stranded plasmid DNA cleavage assay by *Ttd*Ago. **(A)** Schematic overview of pUC19 plasmid. Black polylines indicate target sites. **(B)** Cleavage assay of *Ttd*Ago in the absence of guides under different concentrations of NaCl. **(C)** Schematic of sequences of the 5′-phosphorylated DNA guides and target plasmid region. The blue region indicates the target region. The red region indicates the sequences of FW or RV guides. Predicted cleavage sites are indicated with a black triangle. **(D)** Plasmid cleavage assay was performed by loading *Ttd*Ago in the reaction buffer containing 10 mM Mg^2+^ with the indicated 5′-P-gDNAs at 37°C for 10 min, followed by incubation with the target plasmid at 75°C for 20 min. The analysis of the target plasmid was performed by electrophoresis. M, 5,000 bp DNA ladder; linear, linearized plasmid; SC, supercoiled plasmid; OC, open-circular plasmid.

Previous studies showed that pAgos can not only use gDNA to cleave ssDNA specifically but can also cleave plasmid DNA targets *in vitro* under the guidance of a complementary pair of DNA guides to generate double-stranded (dsDNA) breaks, or in a guide-independent manner (Swarts et al., 2014a; Swarts et al., 2015; Zander et al., 2017; Liu Y. et al., 2021; Guo et al., 2021). We first evaluated dsDNA cleavage in the absence of gDNA. When *Ttd*Ago was incubated with plasmid pUC19 (Figure 6A) at 75°C in the reaction buffer with 5 mM Mn^2+^ for 20 min, there were no cleavage products observed and target plasmids were basically completely degraded (Supplementary Figure S5). Considering the facts that *Ttd*Ago has more than 50% sequence similarity with *Pf*Ago and the presence of Mn^2+^ results in the degradation of plasmid in the plasmid cleavage assay of *Pf*Ago (Swarts et al., 2015), we replaced the divalent metal ions in the reaction buffer and performed the assay at 75°C in the reaction buffer containing 10 mM Mg^2+^ for 20 min. Our result exhibited that the plasmid generated a small amount of open-circular plasmid even in the absence of *Ttd*Ago in buffer with 100 mM NaCl (Figure 6B).

Surprisingly, when *Ttd*Ago was added, a linearized plasmid was detected even in the absence of guides, but that was not observed when *Ttd*Ago_DM was used. These findings suggested that this guide-independent non-specific plasmid relaxation is caused by *Ttd*Ago, which is consistent with previous plasmid cleavage experiments using pAgos from other thermophilic organisms such as *Tt*Ago, *Pf*Ago, *Mj*Ago, and *Tps*Ago (Swarts et al., 2014a; Swarts et al., 2015; Zander et al., 2017; Sun et al., 2022). Fang et al. (2022) predicted the structure of *Ttd*Ago using the AlphaFold web tool and proposed that the cavity of *Ttd*Ago could freely bind to dsDNA when the guide is not loaded, and then dsDNA could be cleaved by the conserved DEDH catalytic center. Furthermore, when the assay was performed in a buffer with 250 mM NaCl, the guide-free *Ttd*Ago-mediated cleavage was not observed. These results indicated that this guide-independent non-specific degradation of plasmid DNA decreased with increasing NaCl concentration.

We then investigated whether *Ttd*Ago can utilize gDNA to cleave plasmid DNA. A pair of 18-nt 5′-P-gDNAs (Supplementary Table S1) named forward and reverse guides (“FW”’ and “RV” guides, respectively) were designed corresponding to the same target region of the pUC19 plasmid. In addition, two non-complementary guides (“NC” guides) were also designed with a random sequence with no overlap with pUC19 (Figure 6C). *Ttd*Ago and gDNAs were incubated with pUC19 in buffers containing 100 or 250 mM NaCl, and both *Ttd*Ago complexes with FW and RV guides were mixed and incubated with pUC19. Compared to the reaction with no guides or NC guides, the linearized plasmid was only detected in the assay containing FW or RV guides (Figure 6D). Moreover, the cleavage efficiency was higher in the reaction containing both FW and RV guides than that containing either FW or RV guide only. In summary, *Ttd*Ago can target the dsDNA plasmid with gDNA resulting in a dsDNA break.

Like the bacterial *Tt*Ago and archaeal *Pf*Ago (Swarts et al., 2015; Jolly et al., 2020), *Ttd*Ago can utilize a single gDNA or a pair of gDNAs to generate dsDNA breaks, though they do not belong to the same branch in the phylogenetic tree. These findings indicate that there is a high conservation of pAgos in function, and pAgos such as *Ttd*Ago might be used for the development of pAgo-based genome-editing tools.

### Application of *Ttd*Ago in *Z. mobilis*

**FIGURE 7:**
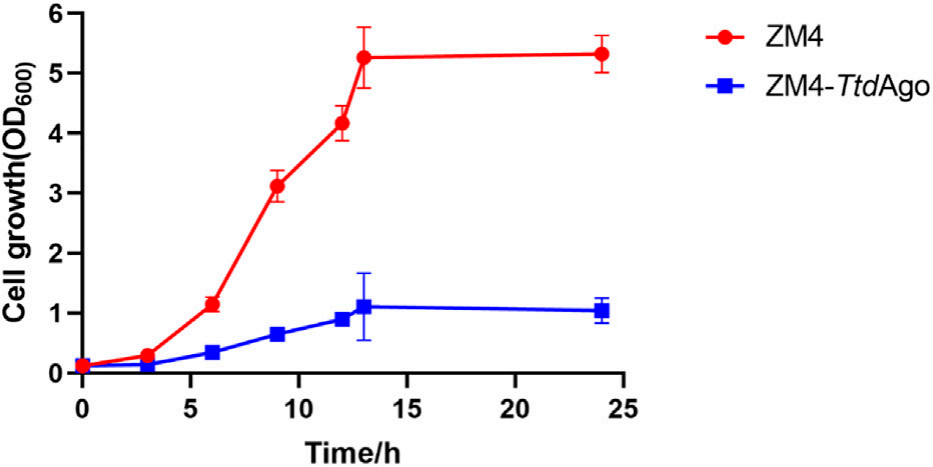
Effect of *TtdAgo* on cell growth of *Zymomonas mobilis*. *Zymomonas mobilis* wild-type (ZM4) strains with *Ttd*Ago expression (ZM4-*Ttd*Ago) were cultured at 30°C. The error bars indicate standard deviations based on three replicates.

To explore the potential of applying *Ttd*Ago to develop pAgo-based genome-editing tools in *Z. mobilis,* the pTZ22b-*TtdAgo* plasmid expressing *Ttd*Ago was constructed and then introduced into *Z. mobilis* to generate the recombinant strain ZM4-*Ttd*Ago. The impact of *Ttd*Ago expression on *Z. mobilis* was then evaluated, and the result indicated that the introduction of *Ttd*Ago reduced the cell growth of ZM4-*Ttd*Ago dramatically. As shown in Figure 7, compared to the wild-type with an OD_600_ value of 5.32 ± 0.12 and growth rate of 0.39, ZM4-*Ttd*Ago had a final OD_600_ value of 1.06 ± 0.07 and growth rate of 0.25. These results indicated that *Ttd*Ago is toxic to *Z. mobilis,* which may be due to the nuclease activity of *Ttd*Ago as reported for the programmable nucleases Cas9 (Pruett-Miller et al., 2009; Morgens et al., 2016) and the capability of *Ttd*Ago to freely bind and cleave dsDNA even when the guide is not loaded (Fang et al., 2022)*.* Therefore, further study is needed to alleviate the cell toxicity of *Ttd*Ago for pAgo-based genome-editing tool development in *Z. mobilis*.

## Conclusion

Our study demonstrated that *Ttd*Ago from the hyperthermophilic archaeon *T. thioreducen*s functions as an endonuclease programmed with gDNAs to cleave both ssDNA and dsDNA plasmids at elevated temperatures, which is similar to the majority of pAgos that have a strong preference for DNA targets. The efficiency and accuracy of cleavage by *Ttd*Ago are modulated by temperature, divalent ions, and the phosphorylation and length of gDNAs and their complementarity to the DNA targets. *Ttd*Ago cleaved DNA at temperatures ranging from 30°C to 95 °C and had good DNA cleavage activity at 70–80 °C with the requirement of Mn^2+^ or Mg^2+^ as cations. In addition, the first attempt to introduce *Ttd*Ago into the industrial microorganism *Z. mobilis* in this study indicated that *Ttd*Ago is toxic to the host, and further study is needed to tune its expression for pAgo-based genome-editing tool development.

## Data Availability

The original contributions presented in the study are included in the article/Supplementary Material; further inquiries can be directed to the corresponding authors.
