# Supplementary material for: In vitro characterization of a pAgo nuclease TtdAgo from Thermococcus thioreducens and evaluation of its effect in vivo
Source: Front Bioeng Biotechnol. 2023 Mar 2;11:1142637. doi: 10.3389/fbioe.2023.1142637 (PMC10017986; doi:10.3389/fbioe.2023.1142637)
Supplement: Supplementary file 1 [file DataSheet1.PDF]

Supplementary Materials

A

Protein	Source	D	E	D	X	Identity（%）
hAgo2	-*Homo sapiens*	FLGADVTHP	QHRQE-IIQD	IFYRDGVSE	AYYAHLVAF	14.78
KpAgo	-*Kluyveromyces Polysporus*	VLGSDVTHY	DGPGEEIITN	MYFRDGVSV	VYYADLLCT	13.61
RsAgo	-*Rhodobacter Sphaeroides*	VVGMGLAEL	EYEGYS---D	VFHAHRPLK	IFYSERIAE	14.21
MbpAgo	-*Mucilaginibacter paludism*	YIGIDVHDR	SQRVEKVRAK	VIVRDGRSF	IKLIDTLLE	15.48
MjAgo	-*Methanocaldococcus jannaschii*	IMGLDTGLG	AP-GERLHLP	LFLRDGFIQ	IHYADKFVK	28.91
PfAgo	-*Pyrococcus furiousus*	IIGIDVAPM	EQRGESVDMN	LLLRDGRIT	VHYAHKFAM	56.36
TtAgo	-*Thermus thermophilus*	AVGFDAGGR	-QAGERIPQE	LLLRDGRVP	LHLADRLVK	18.54
SeAgo	-*Synechococcus elongates*	IIGFDTGTN	-QRGETFSGQ	LLMRDGLVQ	LHLADRSSK	14.40
MpAgo	-*Marinitoga piezophile*	YIGIDLSHD	-ELNEKMNLD	FILRDGRFT	LHIANKVAL	17.21
NgAgo	-*Natronobacterium gregoryi*	FIGIDVSRS	-QLGEKLQST	VIHRDGFMN	TAYADQAST	16.2
LrAgo	-*Limnothrix rosea*	IVGLDVSRR	-IDGEILPEH	LIHRDGLFP	TYYADKIST	15.32
CbAgo	-*Clostridium butyricum*	FIGLDVGTR	-QSGEKIAET	VIHRDGFSR	TGYADKICK	17.9
CbcAgo	-*Clostridium.butyricum CWBI1009*	FIGLDVGTR	-QSGEKIAET	VIHRDGFSR	TGYADKICK	18.23
CpAgo	-*Clostridium perfingens*	FVGLDVGTR	-QNGEKINTE	VIHRDGFSR	TGYADKICK	18.96
IbAgo	-*Intestinibacter bartlettii*	YIGLDVC-R	-QSGEKIQIN	VFHRDGINR	TYYADLSSI	18.34
KmAgo	-*Kurthia massiliensis*	FIGIDVS-H	-LAGEKIDDT	TIHRDGFWR	IHYADLSAT	18.72
MfAgo	-*Methanocaldococcus fervens*	IMGLDSGLG	SP-GERIPIH	LFLRDGFVQ	IHYADKFVK	29.38
FpAgo	-*Ferroglobus placidus*	ILGIDVGYG	YPSKETARIK	LILRDGRIN	IYYADKLVK	24.86
TpsAgo	-*Thermus parvatiensis*	AVGFDAGGR	A--GERIPQE	LLLRDGRVP	LHLADRLVK	19.73
TtdAgo	-*Thermococcus thioreducens*	IVGIDVTPM	EQMGESIDMK	LILRDGKIT	VHYAHKFVR	100
TtdAgo_DM	-*Thermococcus thioreducens mutant*	IVGIAVTPM	EQMGESIDMK	LILRAGKIT	VHYAHKFVR	

B

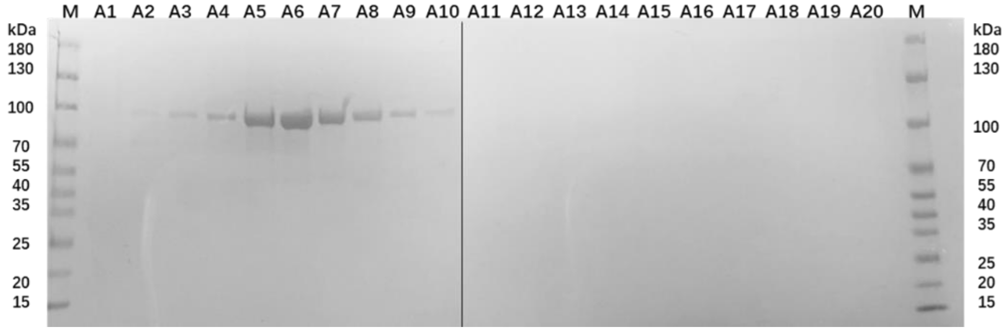

Supplementary Figure S1. Multiple sequence alignment of *Ttd*Ago with Ago proteins **(A)** and SDS-PAGE result of chromatography fractions containing *Ttd*Ago **(B)**. ***Ttd*Ago _DM** is a catalytically dead variant of *Ttd*Ago protein with amino acid substitutions within the catalytic tetrad. **M**: marker; **A2-A10**: chromatography fractions containing *Ttd*Ago.

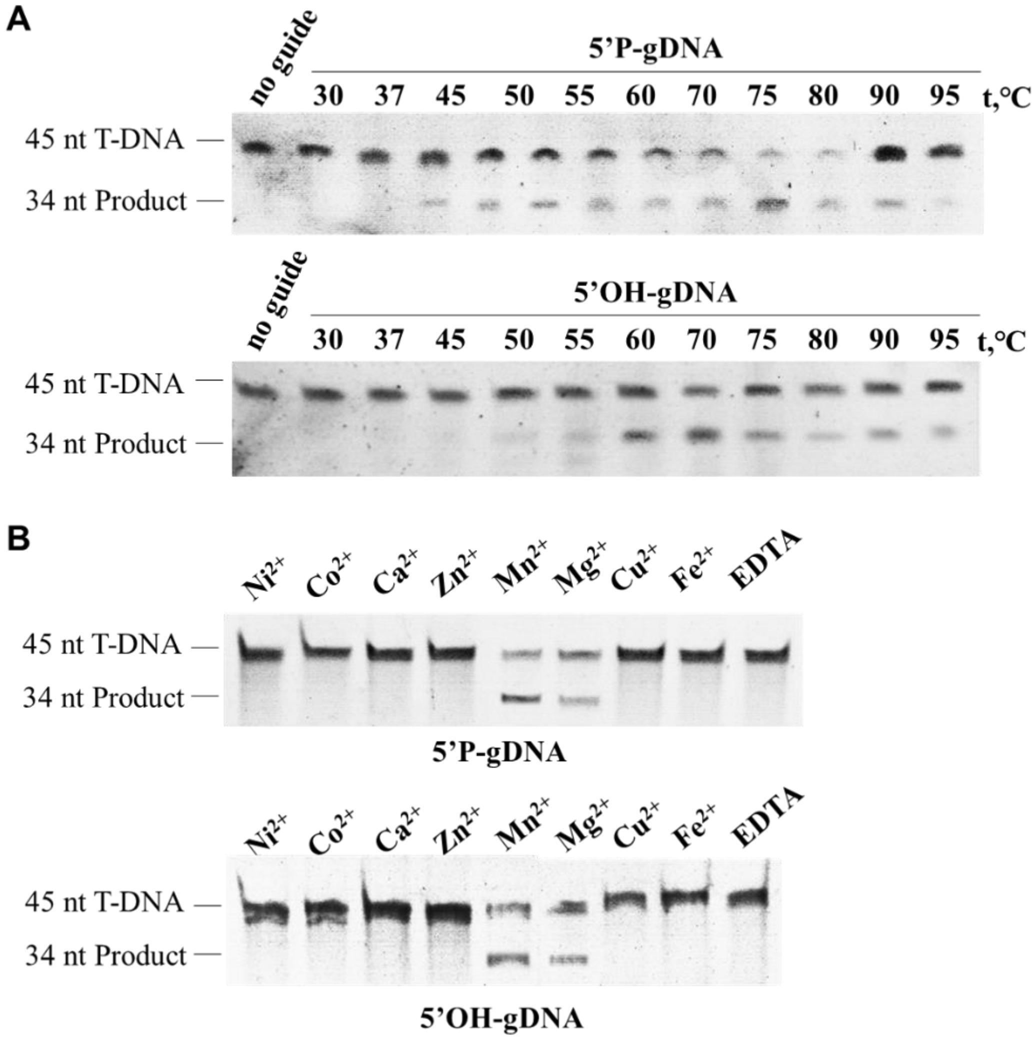

Supplementary Figure S2. Representative denaturing PAGE showing DNA cleavage activity of *Ttd*Ago under different temperatures (A) and different cations (B) using 5'P-gDNA or 5'OH-gDNA. Three independent experiments have been carried out with similar results.

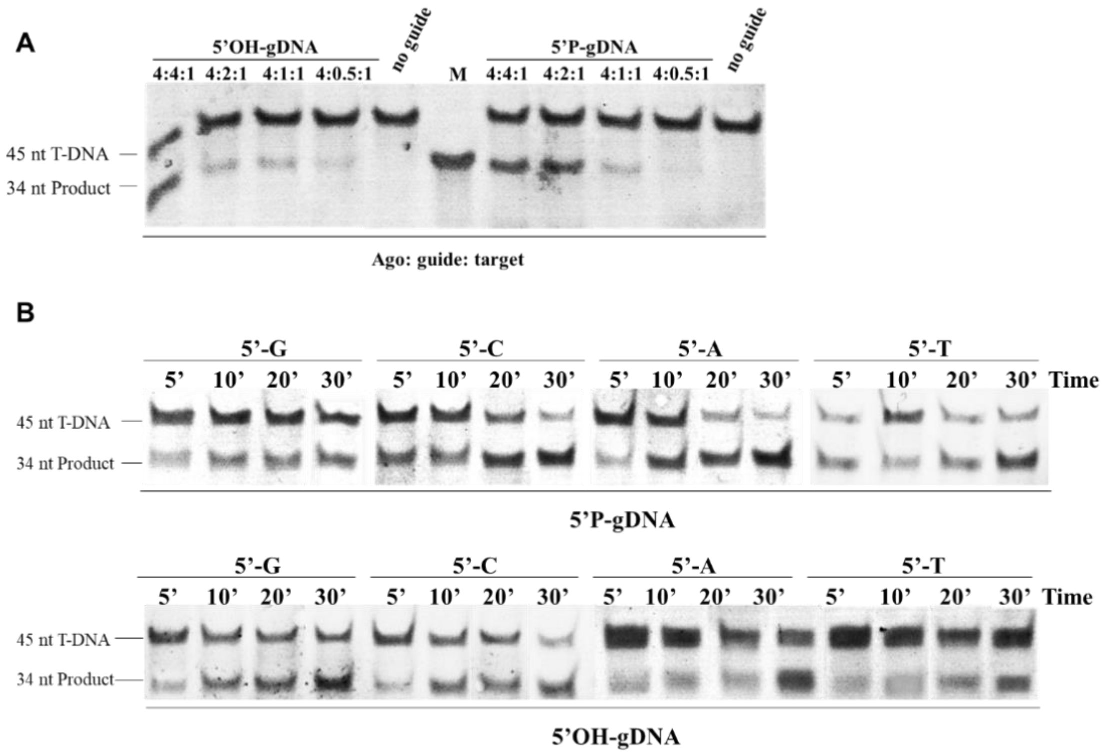

Supplementary Figure S3. Representative SDS-PAGE result showing *Ttd*Ago activity under different guide concentrations (A) or gDNA with different 5'-nucleotide (B) using 5'P-gDNA or 5'OH-gDNA. Time points were taken at 5, 10, 20, 30 min. Three independent experiments have been carried out with similar results.

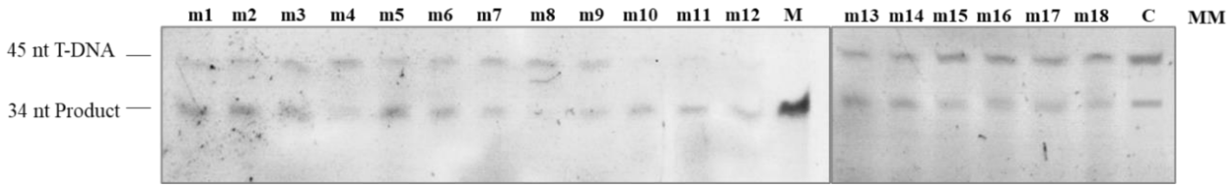

Supplementary Figure S4. Representative SDS-PAGE result showing effects of mismatches in the guide-target duplex on the slicing activity of *Ttd*Ago. M: marker; **C**: control reactions with guide variants containing no substitutions; **MM**: mismatch position. Three independent experiments have been carried out with similar results.

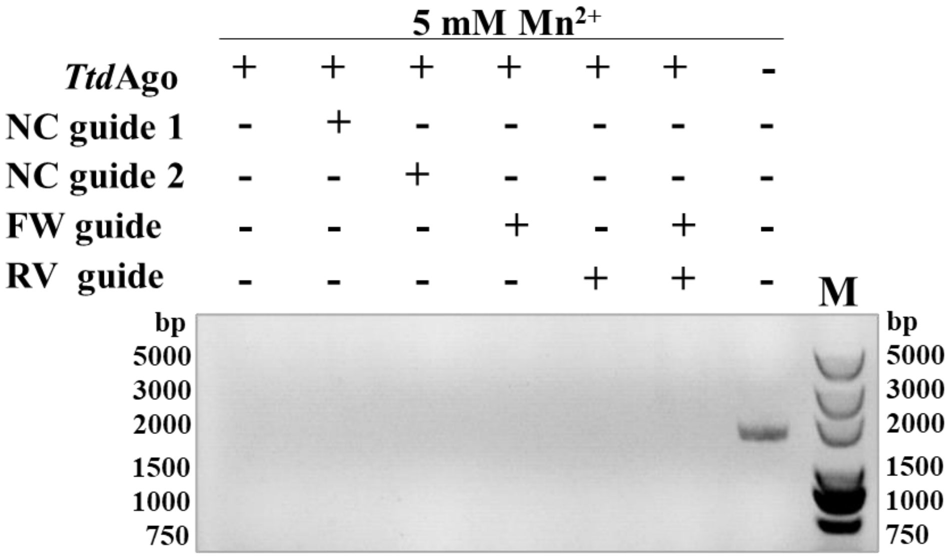

Supplementary Figure S5. Plasmid DNA cleavage assay by *Ttd*Ago in buffer containing 5 mM Mn^{2+}. The experiment was performed by loading *Ttd*Ago with the indicated 5'P-gDNAs at 37 °C for 10 min, followed by incubation with the target plasmid at 75 °C for 20 min. NC, FW and RV guide indicate Non-complementary, forward and reverse guide, respectively. **M**: 5000 bp DNA ladder.

TABLE S1: List of oligonucleotide sequences used in this study

Name	Sequence（5'-3')	Description
U-gRNA	UGAGGUAGUAGGUUGUAU	guide forms 5'-C pair with U-tDNA/U-tRNA
U-tRNA	AAACGACGGCCAGUGCCAAGCUU ACUAUACAACCUACUACCUCAU	45 nt RNA target for U-gDNA/U-gRNA
C-gDNA	CGAGGTAGTAGGTTGTAT	guide forms 5'-C pair with C-tDNA
T-gDNA	TGAGGTAGTAGGTTGTAT	guide forms 5'-T pair with T-tDNA/T-tRNA
A-gDNA	AGAGGTAGTAGGTTGTAT	guide forms 5'-A pair with A-tDNA
G-gDNA	GGAGGTAGTAGGTTGTAT	guide forms 5'-G pair with G-tDNA
A-tDNA	AAACGACGGCCAGTGCCAAGCTT ACTATACAACCTACTACCTCTT	45 nt DNA target for A-gDNA
G-tDNA	AAACGACGGCCAGTGCCAAGCTT ACTATACAACCTACTACCTCCT	45 nt DNA target for G-gDNA
C-tDNA	AAACGACGGCCAGTGCCAAGCTT ACTATACAACCTACTACCTCGT	45 nt DNA target for C-gDNA
T-tDNA	AAACGACGGCCAGTGCCAAGCTT ACTATACAACCTACTACCTCAT	45 nt DNA target for T-gDNA/U-gRNA
34nt DNA product	AAACGACGGCCAGTGCCAAG CTTACTATACAACC	DNA product for 45 nt DNA target
gDNA_mm1	AGAGGTAGTAGGTTGTAT	mismatched at position 1 with T-tDNA
gDNA_mm2	TCAGGTAGTAGGTTGTAT	mismatched at position 2 with T-tDNA
gDNA_mm3	TGTGGTAGTAGGTTGTAT	mismatched at position 3 with T-tDNA
gDNA_mm4	TGACGTAGTAGGTTGTAT	mismatched at position 4 with T-tDNA
gDNA_mm5	TGAGCTAGTAGGTTGTAT	mismatched at position 5 with T-tDNA
gDNA_mm6	TGAGGAAGTAGGTTGTAT	mismatched at position 6 with T-tDNA
gDNA_mm7	TGAGGTTGTAGGTTGTAT	mismatched at position 7 with T-tDNA
gDNA_mm8	TGAGGTACTAGGTTGTAT	mismatched at position 8 with T-tDNA
gDNA_mm9	TGAGGTAGAAGGTTGTAT	mismatched at position 9 with T-tDNA
gDNA_mm10	TGAGGTAGTTGGTTGTAT	mismatched at position 10 with T-tDNA
gDNA_mm11	TGAGGTAGTACGTTGTAT	mismatched at position 11 with T-tDNA
gDNA_mm12	TGAGGTAGTAGCTTGTAT	mismatched at position 12 with T-tDNA
gDNA_mm13	TGAGGTAGTAGGATGTAT	mismatched at position 13 with T-tDNA
gDNA_mm14	TGAGGTAGTAGGTAGTAT	mismatched at position 14 with T-tDNA
gDNA_mm15	TGAGGTAGTAGGTTCTAT	mismatched at position 15 with T-tDNA
gDNA_mm16	TGAGGTAGTAGGTTGAAT	mismatched at position 16 with T-tDNA
gDNA_mm17	TGAGGTAGTAGGTTGTTT	mismatched at position 17 with T-tDNA
gDNA_mm18	TGAGGTAGTAGGTTGTAA	mismatched at position 18 with T-tDNA

11nt T-gDNA	TGAGGTAGTAG	11 nt guide pair with T-tDNA
12nt T-gDNA	TGAGGTAGTAGG	12 nt guide pair with T-tDNA
13nt T-gDNA	TGAGGTAGTAGGT	13 nt guide pair with T-tDNA
14nt T-gDNA	TGAGGTAGTAGGTT	14 nt guide pair with T-tDNA
15nt T-gDNA	TGAGGTAGTAGGTTG	15 nt guide pair with T-tDNA
16nt T-gDNA	TGAGGTAGTAGGTTGT	16 nt guide pair with T-tDNA
17nt T-gDNA	TGAGGTAGTAGGTTGTA	17 nt guide pair with T-tDNA
19nt T-gDNA	TGAGGTAGTAGGTTGTATA	19 nt guide pair with T-tDNA
20nt T-gDNA	TGAGGTAGTAGGTTGTATAG	20 nt guide pair with T-tDNA
21nt T-gDNA	TGAGGTAGTAGGTTGTATAGT	21 nt guide pair with T-tDNA
25nt T-gDNA	TGAGGTAGTAGGTTGTATAGT AAGC	25 nt guide pair with T-tDNA
30nt T-gDNA	TGAGGTAGTAGGTTGTATAGT AAGCTTGGC	30 nt guide pair with T-tDNA
40nt T-gDNA	TGAGGTAGTAGGTTGTATAGT AAGCTTGGCACTGGCCGTC	40 nt guide pair with T-tDNA
NC guide 1	CGAGGTAGTAGGTTGTAT	Noncomplementary guide
NC guide 2	ACTCAAGGCAAGCTTTAT	Noncomplementary guide
FW guide	GGATGTATGGAGCGAGACGAT	guide corresponding to target region of the pUC19 plasmid with a GC-content up to 56%
RV guide	AATCGTCTCGTCCATACATCC	guide corresponding to target region of the pUC19 plasmid with a GC-content up to 56%

TABLE S2 Strains, plasmids, and primers used in this study.

Strains/Plasmids	Description	Reference
**Strain**		
E. coli DH5α	*E. coli* for plasmid construction	Lab stock
E. coli Rosetta (DE3)	*E. coli* for protein expression	Lab stock
Rosetta -*Ttd*Ago	*E. coli* Rosetta (DE3) containing pET23a-His6-*TtdAgo*	This work
Rosetta – *Ttd*Ago_*DM*	*E. coli* Rosetta (DE3) containing pET23a-His6-*TtdAgo_DM*	This work
ZM4	*Z. mobilis* wild-type strain	Lab stock
ZM4-*Ttd*Ago	ZM4 containing pTZ22b-*TtdAgo*	This work
**Plasmid**		
pET23a-His6-*TtdAgo*	pET containing construct His6-*TtdAgo*	This work
pET23a-His6-*TtdAgo_DM*	pET containing construct His6-*TtdAgo_DM*	This work
pTZ22b-*TtdAgo*	pTZ containing construct *TtdAgo*	This work
**Primer**		
DM_D538A_F	GGTATTGCAGTTACCCCGATG	This work
DM-D608A-R	CTTTGGTAATTTTACCTGCACGCAG	This work
*Ttd*Ago_F	GGATCCATGCTGATGAAAGTTCTGAC	This work
*Ttd*Ago_R	CTCGAGAACAAAATACAGAAAACCTTCTGC	This work
